# Society Meetings

**Published:** 1897-09

**Authors:** 


### Society Meetings.

Officers of the Buffalo Academy of Medicine.—Session of 1897–1898.—President, Dr. Lucien Howe; secretary, Dr. Thomas F. Dwyer; treasurer, Dr. E. A. Smith; trustees, Dr. M. Hartwig (three years), Dr. DeLancey Rochester (two years), Dr. Henry R. Hopkins (one year).

The vice-presidents by virtue of being chairmen of the various sections are: Dr. DeLancey Rochester, chairman section on medicine; Dr. Chauncey P. Smith, chairman section on surgery; Dr.

Charles E. Congdon, chairman section on obstetrics; Dr. Frank J. Thornbury, chairman section on pathology.

The council is therefore composed of the aforementioned officers, the president of the Academy of the previous year, Dr. Herman Mynter, and the secretaries of the sections who are: Dr. Wm. G. Ring, secretary section on medicine; Dr. A. E. Diehl, secretary section on surgery; Dr. C. J. Reynolds, secretary section on obstetrics; Dr. J. C. Clemesha, secretary section on pathology.

PROGRAM 1897–98.

September 7th.—Surgery.—Subject to be announced.

September 14th.—Medicine.—Quarterly meeting of Academy.—Medico-legal aspects of cases of poisoning; with especial reference to restriction of sale of poisons, A. L. Benedict, M. D.; Hon. Arthur W. Hickman.

September 21st.—Pathology.—Some observations of the physiology of the stomach; report of X-ray exhibition, A. L. Benedict, M. D. Abnormal cell development in plants and animals; a study in comparative pathology, Miss Mary Forster, Nawnham College, England.

September 28th.—Obstetrics.—Shall we leave the uterus in situ in excision of the adnexa? C. C. Frederick, M. D. Report of three cases of hydrocephalus, L. Schröter, M. D.

October 5th.—Surgery.—Subject to be announced.

October 12th.—Medicine.—Clinical examination of blood, Helena Kuhlman, M. D. Sputum, Nelson G. Russell, M. D.

October 19th.—Pathology.—Recent researches in the pathology of trichophytosis, William Thomas Corlett, M. D., L. R. C. P., Lond., professor of dermatology and syphilology Western Reserve University, Cleveland, O.

October 26th.—Obstetrics.—Subject to be announced. Charles A. L. Reed, M. D., Cincinnati, O. Gonorrhea in women, J. Henry Dowd, M. D.

November 2d.—Surgery.—Subject to be announced.

November 9th.—Medicine.—Renal calculus, John H. Musser, M. D., professor clinical medicine, University of Pennsylvania. Discussion opened by Charles G. Stockton, M. D., and Herman Mynter, M. D.

November 16th.—Pathology.—Skin-grafting, history, physiology and indications with account of a new and original method, Zera J. Lusk, M. D., Warsaw, N. Y.

November 23d.—Obstetrics.—Diagnosis and treatment of carcinoma of the cervix, M. A. Crockett, M. D.

December 7th.—Surgery.—Quarterly meeting of Academy.—Subject to be announced.

December 14th.—Medicine.—The Jew in medicine; a historical sketch, Julius Ullman, M. D.

December 21st.—Pathology.—The possible transmission of disease by wearing "second-hand" clothing, with record of experiments, William G. Bissell, M. D. Gangrenous appendicitis complicated by portal phlebitis and multiple hepatic abscess, Earl P. Lothrop, M. D.

December 28th.—Obstetrics.—Appendicitis in its relation to obstetrics, Eugene Smith, M. D.

January 4th.—Surgery.—Subject to be announced. Roswell Park, M. D.

January 11th.—Medicine.—Pulmonary disturbance in children; infantile asthma, Irving M. Snow, M. D. Empyema, Eugene A. Smith, M. D.

January 18th.—Pathology.—Toxic degeneration of the nervous system, Ira Van Gieson, M. D., director Pathological Institute, New York State commission in lunacy, New York.

January 25th.—Obstetrics.—Subject to be announced. James F. W. Ross, M. D., Toronto, Ont.

February 1st.—Surgery.—Subject to be announced. W. C. Phelps, M. D.

February 8th.—Medicine.—Pulmonary Tuberculosis: 1. Early diagnosis, F. J. Thornbury, M. D.; 2. Prognosis and treatment, S. S. Green, M. D.

February 15th.—Pathology.—Multiple cerebro-spinal sclerosis, illustrated, Floyd S. Crego, M. D.

February 22d.—Obstetrics.—Etiology, pathology, diagnosis and treatment of myofibroma of uterus, H. D. Ingraham, M. D.

March 1st.—Surgery.—Subject to be announced. Dr. H. Mickle.

March 8th.—Medicine.—Diet in Bright's disease, F. C. Shattuck, M. D., professor clinical medicine, Harvard. Discussion opened by Allen A. Jones, M. D.

March 15th.—Pathology.—Quarterly meeting of Academy.—The anatomy and mechanism of the foot, considered with reference to certain abnormal conditions, L. A. Weigel, M. D., Rochester, N. Y.

March 22d.—Obstetrics.—Subject to be announced. L. S. McMurtry, M. D., Louisville, Ky. Asepsis and antisepsis as applied to obstetrics, William Warren Potter, M. D.

April 5th.—Surgery.—Subject to be announced.

April 12th.—Medicine.—Pericarditis ; its diagnosis and its consequence, J. H. Pryor, M. D. Myocardial changes ; early diagnosis and prevention of further development, H. C. Buswell, M. D.

April 19th.—Pathology.—The pathology of the cerebellum, William C. Krauss, M. D. Some pathological findings in the blood ; exhibition of slides, Albert E. Woehnert, M. D.

April 26th.— Obstetrics.— Etiology, pathology, diagnosis and treatment of acute salpingitis, William G. Taylor, M. D. The bacteriology of the female genital tract in health and disease. Frank J. Thornbury, M. D.

May 3d.—Surgery.—Subject to be announced. Eugene Smith, M. D., W. H. Heath, M. D.

May 10th.—Medicine.—Municipal hygiene, H. R. Hopkins, M. D., W. G. Bissell, M. D.

May 17th.—Pathology.—An experimental study of the effect of occlusion of the ureters upon the kidneys, William L. Baum, M. D., professor of skin and venereal diseases, Post-graduate Medical School, Chicago, Ill.

May 24th.—Obstetrics.—Conservative treatment of the ovary, Herman E. Hayd, M. D. Subject to be announced. B. C. Johnson, M. D.

June 7th.—Surgery.—Subject to be announced.

June 14th.—Annual meeting of Academy.

June 21st.—Pathology.—Subject to be announced.

June 28th.—Obstetrics.—Quarterly meeting of Academy.—Subject to be announced. Joseph Price, M. D., Philadelphia, Pa.

---

The Mississippi Valley Medical Association will hold its next annual meeting at Louisville, October 5, 6, 7 and 8, 1897. The executive committee met recently, at Louisville, in conjunction with the local committee of arrangement, the following being present : Drs. Stucky, Grant, Mathews, Love, Holloway and Reynolds. It is expected to make the coming meeting the largest and best in the history of the Association, and everything points to a fulfillment of this endeavor. The railroads will make a round-trip rate of one and a third fare. The address on surgery will be delivered by Dr. J. B. Murphy, Chicago; the address on medicine by Dr. John V. Shoemaker, Philadelphia. The following papers have been offered: I. A. Abt, Chicago, The nature of croup following measles; J. C. Ayers, Cincinnati, Further observations in the use of hydrogen dioxide in the treatment of blepharitis marginalis; W. F. Barclay, Pittsburg, Milk: its production and uses; J. F. Barnhill, Indianapolis, Regarding hypertrophied faucial tonsils; J. M. Batten, Pittsburg, Report of five cases of heart disease; J. K. Bauduy, St. Louis, Some new thoughts in the treatment of locomotor ataxia; A. C. Bernays, St. Louis, Paper; A. F. Bock, St. Louis, The surgical treatment of Basedow's disease; John Young Brown, St. Louis, Some remarks on appendicitis; Sanger Brown, Chicago, Some anomalous conditions of the spinal cord, with report of cases; Eug. G. Carpenter, Cleveland, Posterior radicular neuritis; W. Cheatham, Louisville, Of what assistance has the serum treatment of diphtheria been to the general practitioner? Archibald Church, Chicago, The differential diagnosis and treatment of cerebral hemorrhage and cerebral softening; J. W. Cokenower, Des Moines, Ia., Neurotic deformities in children; A. H. Cordier, Kansas City, Ectopic pregnancy, clinical and pathologic phases; J. Homer Coulter, Chicago, Paper; Ephraim Cutter, New York, Beef—a war paper; Richard Dewey, Wauwatosa, Wis., Some cases of insanity in adolescence; Arch Dixon, Henderson, Ky., To drain or not to drain; Kennon Dunham, Cincinnati, The hypodermic syringe and its use in malaria; C. Travis Drennan, Hot Springs, Ark., Report of a case of anesthesia produced by mercury, with remarks; Sherwood Dunn, Los Angeles, Mothers and daughters; J. Rilus Eastman, Indianapolis, Diagnosis by inspection in the urinary tract; A. R. Edwards, Chicago, The diagnosis of abscess of the liver based upon a study of twenty-five cases; Jos. Eichberg, Cincinnati, Typhoid fever treated without cold baths; C. Fisch, St. Louis, The antitoxic and bactericidal properties of the serum of horses treated with Koch's new tuberculin (T. R.); F. R. Fry, St. Louis, Pressure symptoms after head injuries; A. H. Goelet, New York, The surgical treatment of fibroid tumors of the uterus; Spencer Graves, St. Louis, Appendicitis; H. Hatch, Quincy, Ill., Severe injuries from electricity, and what best to do; A. G. Hobbs, Mouth-breathing in children; Discussion opened by Dr. H. W. Loeb; B. W. Holliday, Cleveland, The civic aspect and therapy of some of the common neuroses; A. F. House, Cleveland, Symptoms and surgical treatment of perforated intestinal ulcers ; W. H. Humiston, Cleveland, Cocaine anesthesia in perineorrhaphy ; C. C. Jacobs, Frostburg, Md., The treatment of obstructive lesions of the urinary tract, anterior to the bladder, with especial reference to the enlargement of the prostate gland ; A. C. Klebs, Chicago, Paper ; E. L. Larkins, Terre Haute, Ind., Appendicitis ; F. F. Lawrence, Columbus, O., Hysterectomy ; Elmer Lee, New York, The elimination of empiricism in the treatment of pneumonia ; I. N. Love, St. Louis, The relations of the secular press to medicine and the public ; C. F. McGahan, Aiken, S. C., The treatment of pulmonary phthisis ; A. H. Meisenbach, St. Louis, A plea for early operation in cholelithiasis ; L. Harrison Mettler, Chicago, Neuroses of gout ; Robt. T. Morris, New York, Paper ; Harold N. Moyer, Chicago, Paper ; A. M. Owen, Evansville, Ind., Cathartics and constipation ; A. J. Ochsner, Chicago, Treatment of hernia in old men ; Curran Pope, Louisville, Ky., Sanitoriums a necessary factor in the treatment of chronic diseases ; Joseph Price, Philadelphia, Paper ; J. Punton, Kansas City, The growing needs of medical political organisation ; D. C. Ramsey, Mt. Vernon, Ind., Municipal sanitation of tuberculosis ; A. Ravogli, Cincinnati, Tuberculin in dermatology ; B. Merrill Rickets, Cincinnati, Abdominal incision for ascites ; Byron Robinson, Chicago, The classification of peritonitis ; Enno Sander, St. Louis, The Carlsbad Springs of the United States of North America ; E. W. Saunders, St. Louis, Therapeutic properties of infant foods ; E. J. Senn, Chicago, The treatment of suppurating fistulous tracts ; E. B. Smith, Detroit, Experimental surgery ; J. O. Stillson, Indianapolis, Retro-bulbar optic neuritis ; L. Strauss, St. Louis, Primary tuberculosis of the rectum with report of cases; J. A. Stucky, Lexington, Ky., Intratympanic surgery in chronic suppuration ; J. B. Taulbee, Mt. Sterling, Ky., The treatment of wounds by the open method ; H. M. Thomas, Chicago, Experimental work on the penetrability of vaporised medicaments in the air-passages ; K. K. Wheelock, Fort Wayne, Ind., Plastic operation for reforming interpalpebral space ; Alex. C. Wiener, Chicago, Congenital dislocation of the hip ; Frank Woodbury, Philadelphia, Paper. Further titles of papers should be sent to Dr. Thomas Hunt Stucky, president, Louisville, or to Dr. H. W. Loeb, secretary, St. Louis, Mo.

---

THE Central New York Medical Association will hold its thirtieth annual meeting at Buffalo, on Tuesday, October 17, 1897.

This association calls together the most progressive physicians of Central and Western New York, and has become widely known as a thorough and scientific working body. The Buffalo meeting promises to be a most successful and important one and should receive the coöperation of all progressive physicians in this and neighboring counties. All members of county societies within the jurisdiction of the association are eligible to membership after attending three consecutive meetings and the payment of $2 initiation fees. The following counties comprise the association: Onondaga, Cayuga, Seneca, Ontario, Wayne, Monroe, Chenango, Cortland, Genesee, Livingston, Madison, Orleans, Oswego, Wyoming, Yates and Erie. There is no reason why Niagara, Cattaraugus, Chautauqua and Allegheny counties should not join the association, and then Western New York would be fully represented. Erie county thus far has been feebly represented, only twenty members belonging to the association, while Monroe has more than double this number of members. The annual dues amount to $1, entitling the members to the volume of transactions.

Titles of papers to be read at the Buffalo meeting should be sent before October 1st to Dr. Edward B. Angell, president, 294 Alexander street, Rochester, N. Y., or to Dr. C. A. Van Der Beek, secretary, Rochester, N. Y., or may be left at this office. In our next issue we hope to publish the preliminary program.

---

THE American Microscopical Society held its twentieth annual meeting at Toledo, O., August 5th, 6th and 7th. Papers of great importance were read by some of the members and a microscopical exhibition was held on August 6th. The address of the president, Prof. E. W. Claypole, of Akron, O., on Microscopic light in geological darkness, was an able and masterful delineation of the value of the microscope in the study of rocks. Another very interesting paper was read by Mr. Francis Scott Rice, of Steelton, Pa., on the Micro-structural characteristics of steel; another, on the Comparison of the phagocytic action of leucocytes in amphibians and mammals, by John M. Berry, Peterboro, N. Y., and one on the Comparative structure of the digestive tract, by Edith J. Claypole, Wellesley, Mass., attracted attention. On Friday afternoon the society was given a boat ride on the Maumee river and Lake Erie, which was thoroughly enjoyed by all who had the good fortune to attend. Syracuse was chosen as the next meeting place and the following were elected officers for 1897–'98: President, Prof. D. S. Kellicott, Ph. D.,

Columbus, O.; vice-presidents, Moses C. White, M. D., New Haven, Conn., and Veranus A. Moore, M. D., Ithaca, N. Y.; secretary, William C. Krauss, M. D., Buffalo, N. Y.; treasurer, Mr. Magnus Pflaum, Pittsburg, Pa. Elective members of the executive committee: D. E. Hoag, M. D., Toledo, O.; R. Aberdein, M. D., Syracuse, N. Y.; Edith J. Claypole, Ph. D., Wellesley, Mass.

---

The Physicians' Club of Buffalo, N. Y., was organised the early part of last winter. The object of the club is the careful consideration of subjects having to do with the science of medicine and the promotion of social intercourse among its members. The officers during the past year were: president, Dr. Thomas F. Dwyer; vice-president, Dr. A. E. Diehl; secretary-treasurer, Dr. Chas. E. Congdon.

At the last meeting, held at the house of Dr. Thomas B. Carpenter, 357 Pennsylvania street, the retiring president, Dr. Thomas F. Dwyer, read a paper on Twin pregnancy, after which the following officers were elected and installed for the coming year: president, Dr. A. E. Diehl; vice-president, Dr. E. M. Dooley; secretary-treasurer, Dr. J. J. Finerty; council, Drs. W. Scott Renner, D. J. Constantine, Thos. B. Carpenter, J. J. Walsh and J. Henry Dowd.

